# Effects of fermented ramie feed on the growth performance, serum biochemistry, metabolic capacity, antioxidant capacity, and intestinal health of *Linwu* ducks

**DOI:** 10.3389/fvets.2025.1646055

**Published:** 2025-08-04

**Authors:** Zonghao Lv, Jingmeng Zhang, Cong Li, Zhen Li, Zhizhong Zhang, Biguang Lv, Xuan Cheng, Haohan Zhao, Qinghua Chen, Qian Lin

**Affiliations:** ^1^College of Animal Science and Technology, Hunan Agricultural University, Changsha, China; ^2^Institute of Bast Fiber Crops, Chinese Academy of Agricultural Sciences, Changsha, China; ^3^Livestock and Fisheries Research Institute, Huaihua Academy of Agricultural Science, Huaihua, China; ^4^Hunan Provincial Key Laboratory of the Traditional Chinese Medicine Agricultural Biogenomics, Changsha Medical University, Changsha, China; ^5^Key Laboratory of Swine Nutrition and Feed Science of Fujian Province, Aonong Group, Zhangzhou, China

**Keywords:** antioxidant capacity, duck, fermented ramie, growth performance, intestinal health, metabolic capacity

## Abstract

This study investigated the effects of fermented ramie feed (FRF) on the growth performance, serum biochemistry, metabolic capacity, antioxidant capacity, and intestinal health of *Linwu* ducks. A total of 480 female *Linwu* ducks (age: 28 days) were randomly divided into five treatments (six replicates per group; 16 ducks per replicate). The control group received a basal diet and the treatment groups received the basal diet supplemented with by 3%, 6%, 9% or 12% FRF. The experiment lasted 21 days. Notably, 3% and 6% FRF trended to increase the final body weight (*P* = 0.097) and significantly increased the thymus index (*P* < 0.05). Regarding serum indices, FRF greatly reduced the triglyceride and glucose contents, and increased the catalase and glutathione peroxidase activities (*P* < 0.05). Besides, FRF significantly increased the apparent metabolic rates of dry matter, crude protein, crude ash and gross energy (*P* < 0.05). Furthermore, FRF remarkably improved villus height in the duodenum. FRF also increased the relative abundances of beneficial bacteria (*Alistipes* and *Barnesiella*) and reduced the relative abundances of harmful parasitic bacteria (*Desulfovibrio* and *Enterenecus*) in the cecum (*P* < 0.05). Thus, partial FRF substitution greatly improved serum biochemistry, antioxidant capacity, metabolic capacity and intestinal health in *Linwu* ducks, thereby enhancing the growth performance to a certain extent. The optimal level of FRF substitution is 3%−6% according to the impacts of growth performance and overall health.

## 1 Introduction

The rapid growth of the livestock industry has been accompanied by increases in the demand for feed ingredients and strains on feed resources (1). Moreover, drastic price fluctuations of key feed ingredients, such as corn and soybean meal (the main components of traditional feed formula in the international market), have hindered the sustainable development of poultry production (2, 3). Under such situation, the Chinese government issued a policy aimed at promoting the development of novel feed resources and optimizing the composition of poultry feed to reduce the dependence on the corn and soybean meal (4). Consequently, identification of high-quality, high-yield alternative feed sources has become a key research focus in poultry nutrition.

Ramie (*Boehmeria nivea*), commonly referred to as “China grass,” is a perennial plant belonging to the Urticaceae family (5). This plant has a nutritional profile similar to that of alfalfa, with juvenile stems and foliage serving as rich source of protein, essential vitamins, and balanced amino acids (6). Additionally, phytochemical analyses have revealed that ramie roots and leaves contain variety of bioactive compounds, particularly flavonoids (e.g., rutin and rofilin) and phenolic acids (e.g., chlorogenic and ferulic acid derivatives), that exhibit antibacterial, anti-inflammatory, and antioxidant properties (7, 8). Thus, ramie holds promise as a feed material. Numerous studies have confirmed the beneficial effects of ramie in poultry production (9–11). A study reported that dietary supplementation with ramie powder increases laying rate and liver antioxidant enzyme activity in hens and protected hen livers from oxidative damage (9). Regarding poultry products, dietary ramie supplementation improved egg yolk quality in laying hens (12) and enhanced thigh muscle meat quality in geese (11). Moreover, it was reported that ramie powder addition enhanced the growth performance and antioxidant capacity and improved meat quality of *Linwu* ducks (10).

Although these findings confirmed the benefits of ramie, the presence of cellulose and anti-nutritional factors in ramie hindered its application in feed (13). Microbial fermentation of feed ingredients can degrade macromolecular substances such as cellulose and antinutritional factors, thus improving feed quality (14, 15). In the processing of soybean meal, microbial fermentation improves not only feed quality but also production performance, gut microbiota homeostasis, and immune function (16, 17). However, few studies have analyzed the effects of fermented ramie feed (FRF) on poultry production. Therefore, the study firstly investigated the effects of FRF on the growth performance, serum biochemistry, metabolic capacity, antioxidant capacity, intestinal morphology and intestinal microbiome of *Linwu* ducks.

## 2 Materials and methods

### 2.1 Preparation of FRF

After harvesting, ramie was chopped into 2-cm segments by using a hay cutter and mixed with wheat bran at a 1:1 ratio for fermentation. The fermentation agent, provided by Yuyi Jiayi Biotechnology (Puyang, China), primarily comprised lactic acid bacteria, yeast, *Bacillus* spp., and their metabolites, with a viable bacterial count of 2 × 10^10^ CFU/g. The fermentation solution was prepared by dissolving the agent (1 kg) in a solution of brown sugar (1 kg) and water (10 kg), which was based on the unpublished data of fermentation process optimization that maximized the nutritional value of ramie. After 6-h of activation at room temperature, 1 mL of the fermentation solution was added per kilogram of the ramie–wheat bran mixture. Fermentation was conducted for 90 days. The resultant FRF (air dry) contained the following ingredients: crude protein (19.20%), crude fat (3.89%), crude fiber (10.29%), and crude ash (6.83%).

### 2.2 Experimental design and diets

The study protocol was approved by the Animal Care Committee of the Institute of Bast Fiber Crops, Chinese Academic of Agricultural Sciences. A total of 480 female *Linwu* ducks (age: 28 days) with similar body weight (BW) were randomly divided into five treatments (six replicates per group; 16 ducks per replicate). The control group received a basal diet, and the treatment groups received a basal diet supplemented with 3%, 6%, 9%, or 12% FRF [Reda et al. (18) for dose setting] to replace the same proportion of basal diet. The basal diet was formulated in accordance with NRC (43) for ducks. The composition and nutrient contents of the diet are detailed in Supplementary Table S1. The feeding trial lasted 21 days, during which feed and water were provided ad libitum. BW was recorded per replicate on days 28 and 49. Feed consumption was measured per replicate to calculate average daily feed intake (ADFI), average daily gain (ADG), and feed to BW gain ratio (F/G).

### 2.3 Sample collection

Feed and fecal samples were collected before the end of the trial to evaluate apparent nutrient metabolic rates. At the end of the trial, one duck with a BW close to the replicate's average was selected, starved for 12 h, and weighed. All selected ducks were exsanguinated after electrical stunning. Wing blood samples were collected and centrifuged at 3,000 × g for 10 min at 4°C to obtain serum, which was stored at −20°C before further analysis. The heart, liver, spleen, pancreas, thymus, bursa of Fabricius, gizzard proventriculus, and intestine were removed and weighed. Organ indices were calculated relative to BW (g/kg). Sections of the mid-duodenum, mid-jejunum, and mid-ileum were fixed in 4% paraformaldehyde for morphological evaluation. Cecal contents were collected and stored at −80°C for gut microbiome analysis.

### 2.4 Nutrient metabolism

Acid insoluble ash (AIA) was used as an internal parameter to determine apparent metabolic rate, as described by Zhu et al. (19). This rate was calculated using the following formula: apparent metabolic rate (%) = 100 – A1/A2 × F2/F1 × 100, where A1 means the feed AIA content, A2 means the fecal AIA content, F1 means the feed nutrient content, and F2 means the fecal nutrient content.

### 2.5 Serum biochemistry

Serum concentrations of total protein (TP), albumin (ALB), globulin (GLB), blood urea nitrogen (BUN), total cholesterol (TC), triglyceride (TG), glucose (GLU), aspartate aminotransferase (AST), alanine aminotransferase (ALT), alkaline phosphatase (ALP), and lactate dehydrogenase (LDH) were measured using a fully automatic biochemical analyzer (URIT−8000 system; URIT Medical Electronic, Guilin, China). Concentrations of high-density lipoprotein cholesterol (HDL-C) and low-density lipoprotein cholesterol (LDL-C) were determined by commercial kits (Nanjing Jiancheng Biochemistry, Nanjing, China).

### 2.6 Serum antioxidant capacity

Serum malondialdehyde (MDA) content, and the activities of total antioxidant capacity (T-AOC), superoxide dismutase (SOD), catalase (CAT), glutathione (GSH), and glutathione peroxidase (GSH-Px) in serum were determined using commercial kits (Nanjing Jiancheng Biochemistry, Nanjing, China) following the manufacturer's instructions.

### 2.7 Intestinal morphology

Intestinal tissue specimens were dehydrated, paraffin-embedded, and stained with hematoxylin and eosin. Villus height (VH) and crypt depth (CD) were observed using an Olympus microscope (Olympus Corporation, Tokyo, Japan) and determined by the CaseViewer (Wuhan servicebio technology, Wuhan, China) to calculate the ratio of VH to CD (V/C).

### 2.8 DNA extraction and 16S rRNA sequencing

Cecal samples were sent to Shanghai Personal Biotechnology (Shanghai, China) for gut microbiome analysis. DNA was extracted from each sample, and its quality was verified before 16S rRNA sequencing. The V3–V4 region was amplified using barcoded primers (forward: 5′-ACTCCTACGGGAGGCAGCA-3′; reverse: 5′-GGACTACHVGGGTWTCTAAT-3′). The amplicons were quantified using the Quant-iT PicoGreen dsDNA Assay Kit. Sequencing libraries were prepared using the Illumina TruSeq Nano DNA LT Library Prep Kit. Qualified libraries were subjected to paired-end sequencing (2 × 250 bp) on an Illumina NovaSeq platform with the NovaSeq 6,000 SP Reagent Kit (500 cycles). Raw sequences were processed using the mothur pipeline [version 1.39.5; (41)], following MiSeq standard operating procedures (https://www.mothur.org/wiki/MiSeq_SOP). Denois sequences were taxonomically classified using data from the Greengenes database (verdion 13_8) and clustered into operational taxonomic units (OTUs) at 97% similarity. Downstream bioinformatics analyses were performed using R (42).

### 2.9 Statistical analysis

Data were analyzed using one-way ANOVA procedure by SPSS18.0 statistical software (SPSS, Inc., Chicago, IL, USA) and expressed as mean ± standard error. Each replicate served as the experimental unit for growth performance, and individual duck served as the experimental unit for other indicators. The linear and quadratic impacts of FRF addition levels were checked by orthogonal polynomial contrasts. *P* < 0.05 indicated significant difference.

## 3 Results

### 3.1 Growth performance

Growth performance data are indicated in Table 1. No significant differences in the final BW, ADFI, ADG, or F/G ratio were noted among the groups (*P* > 0.05). Compared to the control group, 3% and 6% FRF addition tended to increase the final BW (*P* = 0.097).

**Table 1:** Effect of FRF on the growth performance of ducks.

**Items**	**FRF dosage addition**					***P-value***		
	**0**	**3%**	**6%**	**9%**	**12%**	**ANOVA**	**Linear**	**Quadratic**
Initial BW, g	674.50 ± 4.88	679.91 ± 0.82	678.78 ± 2.20	679.63 ± 0.53	673.08 ± 3.35	0.343	0.734	0.058
Final BW, g	1227.16 ± 17.91	1293.21 ± 35.38	1266.62 ± 10.25	1215.08 ± 19.09	1215.09 ± 22.60	0.097	0.173	0.083
ADG, g	26.32 ± 1.08	29.20 ± 1.71	27.99 ± 0.57	25.50 ± 0.91	25.81 ± 1.09	0.155	0.207	0.150
ADFI, g	139.13 ± 3.30	132.69 ± 4.74	136.41 ± 1.71	132.97 ± 2.55	133.77 ± 4.92	0.695	0.381	0.600
F/G	5.30 ± 0.11	4.59 ± 0.31	4.88 ± 0.13	5.22 ± 0.09	5.20 ± 0.25	0.109	0.487	0.072

FRF, fermented ramie feed; BW, body weight; ADFI, average daily feed intake; ADG, average daily gain; F/G, ratio of feed intake to body weight gain.

### 3.2 Organ index

Data for organ index are shown in Table 2. No obvious between-group differences were moted in the organ index of the heart, liver, pancreas, bursa of Fabricius, spleen, gizzard, proventriculus or intestine among groups were noted among the groups (*P* > 0.05). Notably, 3%, 6%, 9%, and 12% FRF addition significantly increased the thymus index (linear and quadratic, *P* < 0.05).

**Table 2:** Effect of FRF on organ indices of ducks.

**Items**	**FRF dosage addition**					***P-value***		
	**0**	**3%**	**6%**	**9%**	**12%**	**ANOVA**	**Linear**	**Quadratic**
Heart, %	0.74 ± 0.03	0.71 ± 0.02	0.66 ± 0.03	0.70 ± 0.02	0.73 ± 0.03	0.235	0.636	0.034
Liver, %	2.17 ± 0.0 5	2.05 ± 0.05	2.15 ± 0.10	1.96 ± 0.04	2.18 ± 0.10	0.175	0.773	0.162
Pancreas, %	0.41 ± 0.02	0.35 ± 0.02	0.34 ± 0.02	0.36 ± 0.01	0.36 ± 0.02	0.079	0.098	0.036
Bursa of Fabricius, %	0.12 ± 0.01	0.18 ± 0.02	0.16 ± 0.02	0.15 ± 0.01	0.15 ± 0.02	0.389	0.692	0.220
Thymus, %	0.15 ± 0.01^b^	0.25 ± 0.02^a^	0.23 ± 0.02^a^	0.27 ± 0.03^a^	0.25 ± 0.03^a^	0.003	0.003	0.034
Spleen, %	0.07 ± 0.01	0.09 ± 0.01	0.10 ± 0.02	0.09 ± 0.01	0.09 ± 0.01	0.678	0.263	0.342
Gizzard, %	3.33 ± 0.21	3.56 ± 0.12	3.41 ± 0.13	3.25 ± 0.16	3.68 ± 0.28	0.516	0.525	0.597
Proventriculus, %	0.41 ± 0.02	0.42 ± 0.03	0.44 ± 0.02	0.36 ± 0.01	0.39 ± 0.02	0.174	0.122	0.490
Intestine, %	3.32 ± 0.09	3.62 ±0.13	3.44 ± 0.15	3.24 ± 0.06	3.43 ± 0.12	0.198	0.659	0.602

Means (n = 6) within a row with different superscript letters differ significantly (P < 0.05). FRF, fermented ramie feed.

### 3.3 Serum biochemistry

Data for serum biochemistry are presented in Table 3. No significant differences in the contents of TP, ALB, BUN, TC, AST, ALT, ALP, LDH, HDL-C, and LDL-C and the value of ALB/GLB among groups were recorded among the groups (*P* > 0.05). Compared with the control group, 3%, 6%, 9%, and 12% FRF significantly decreased the serum TG content (linear, *P* < 0.05). Ducks in the 12% FRF group recorded the higher GLU content than those in the other groups (linear and quadratic, *P* < 0.05).

**Table 3:** Effect of FRF on serum biochemical indices of ducks.

**Items**	**FRF dosage addition**					***P-value***		
	**0**	**3%**	**6%**	**9%**	**12%**	**ANOVA**	**Linear**	**Quadratic**
TP, g/L	53.11 ± 2.18	47.07 ± 1.26	47.19 ± 1.15	48.08 ± 1.85	52.15 ± 2.80	0.098	0.884	0.008
ALB, g/L	15.5 ± 0.58	14.22 ± 0.38	14.14 ± 0.32	14.37 ± 0.45	15.30 ± 0.52	0.133	0.866	0.011
GLB, g/L	37.61 ± 1.68	32.86 ± 0.93	33.05 ± 0.87	33.71 ± 1.44	36.85 ± 2.28	0.104	0.893	0.009
ALB/GLB	0.41 ± 0.01	0.43 ± 0.01	0.43 ± 0.01	0.43 ± 0.01	0.42 ± 0.01	0.565	0.85	0.136
BUN, mmol/L	0.57 ± 0.07	0.48 ± 0.04	0.41 ± 0.03	0.44 ± 0.03	0.51 ± 0.05	0.170	0.308	0.024
TC, mmol/L	4.77 ± 0.30	4.05 ± 0.21	4.06 ± 0.11	4.16 ± 0.16	4.49 ± 0.17	0.075	0.484	0.008
TG, mmol/L	0.85 ± 0.05^a^	0.70 ± 0.04^b^	0.72 ± 0.05^b^	0.66 ± 0.04^b^	0.67 ± 0.02^b^	0.023	0.006	0.109
GLU, mmol/L	8.03 ± 0.27^a^	7.88 ± 0.20^a^	7.73 ± 0.43^a^	7.94 ± 0.15^a^	6.49 ± 0.24^b^	0.003	0.002	0.039
AST, U/L	80.34 ± 12.61	117.57 ± 22.52	128.04 ± 19.14	117.62 ± 13.61	108.14 ± 9.74	0.312	0.288	0.071
ALT, U/L	35.94 ± 4.73	32.62 ± 3.76	30.49 ± 1.92	35.86 ± 1.40	32.37 ± 2.30	0.669	0.692	0.54
ALP, U/L	432.5 ± 34.14	414.17 ± 35.76	481.8 ± 47.61	413.87 ± 15.42	344.28 ± 21.49	0.092	0.102	0.064
LDH, U/L	1,569.50 ± 151.59	1,571.67 ± 104.31	1,562.83 ± 176.25	1,782.00 ± 71.33	1,709.67 ± 208.21	0.773	0.313	0.89
HDL-C, mmol/L	3.47 ± 0.18	3.16 ± 0.15	3.05 ± 0.11	3.13 ± 0.16	3.20 ± 0.15	0.378	0.246	0.108
LDL-C, mmol/L	2.55 ± 0.20	1.88 ± 0.26	1.96 ± 0.10	1.94 ± 0.12	1.95 ± 0.20	0.091	0.063	0.079

Means (n = 6) within a row with different superscript letters differ significantly (P < 0.05). FRF, fermented ramie feed; TP, total protein; ALB, albumin; GLB, globulin; ALB/GLB, the ratio of albumin to globulin; BUN, blood urea nitrogen; TC, total cholesterol; TG, triglyceride; GLU, glucose; AST, aspartate aminotransferase; ALT, alanine aminotransferase; ALP, alkaline phosphatase; LDH, lactate dehydrogenase; HDL-C, high-density lipoprotein cholesterol; LDL-C, low-density lipoprotein cholesterol.

### 3.4 Antioxidant status

Data for antioxidant status are shown in Table 4. No marked differences in the GSH, SOD, and T-AOC activities and MDA content among groups were recorded among the groups (*P* > 0.05). Compared with the control group, 3% and 12% FRF supplementation significantly increased serum GSH-Px activity of ducks (*P* < 0.05). Moreover, ducks in the 3% group exhibited the higher CAT activity than those in the other groups (*P* < 0.05).

**Table 4:** Effect of FRF on antioxidant capacity of ducks.

**Items**	**FRF dosage addition**					***P-value***		
	**0**	**3%**	**6%**	**9%**	**12%**	**ANOVA**	**Linear**	**Quadratic**
CAT, U/mL	5.52 ± 0.17^bc^	7.61 ± 0.57^a^	6.13 ± 0.13^bc^	5.03 ± 0.16^c^	6.76 ± 0.74^ab^	0.003	0.937	0.834
GSH, μmol/L	18.91 ± 2.48	16.15 ± 1.14	18.36 ± 2.24	15.98 ± 1.18	22.47 ± 1.51	0.105	0.233	0.049
GSH-Px, U/mL	425.83 ± 33.47^c^	668.35 ± 50.15^a^	512.13 ± 61.62^bc^	529.77 ± 46.31^bc^	602.21 ± 18.89^ab^	0.009	0.141	0.328
SOD, U/mL	180.15 ± 6.57	177.79 ± 4.64	159.74 ± 4.34	173.74 ± 10.15	159.40 ± 2.23	0.062	0.028	0.73
T-AOC, mmol/L	0.79 ± 0.04	0.73 ± 0.03	0.72 ± 0.05	0.70 ± 0.01	0.71 ± 0.03	0.386	0.085	0.314
MDA, nmol/mL	4.78 ± 0.54	3.69 ± 0.35	4.43 ± 0.36	4.30 ± 0.33	4.39 ± 0.60	0.546	0.912	0.380

Means (n = 6) within a row with different superscript letters differ significantly (P < 0.05). FRF, fermented ramie feed; T-AOC, total antioxidant capacity; SOD, superoxide dismutase; CAT, catalase; GSH, glutathione; GSH-Px, glutathione peroxidase; MDA, malondialdehyde.

### 3.5 Nutrient utilization

Data for nutrient utilization are shown in Table 5. Compared with the control group, 3%, 6%, 9%, and 12% FRF significantly increased the apparent metabolic rate of dry matter, crude protein, crude ash, and gross energy in ducks (linear and quadratic, *P* < 0.05). Three percentage and 12% FRF addition significantly improved the apparent metabolic rate of crude fat and crude fat of duck (*P* < 0.05). Moreover, ducks in the 6% group exhibited a higher apparent metabolic rate for crude fat than did the control group (*P* < 0.05).

**Table 5:** Effect of FRF on apparent metabolic rate of nutrients of ducks (%).

**Items**	**FRF dosage addition**					***P-value***		
	**0**	**3%**	**6%**	**9%**	**12%**	**ANOVA**	**Linear**	**Quadratic**
Dry matter	71.23 ± 0.76^b^	78.54 ± 1.65^a^	80.26 ± 1.68^a^	77.47 ± 1.09^a^	78.17 ± 0.74^a^	0.001	0.006	0.002
Crude protein	52.76 ± 3.64^b^	63.80 ± 3.57^a^	67.77 ± 3.07^a^	62.54 ± 2.06^a^	64.58 ± 3.56^a^	0.049	0.045	0.040
Crude ash	30.56 ± 2.03^b^	45.88 ± 3.15^a^	49.31 ± 3.38^a^	44.14 ± 2.19^a^	47.88 ± 2.24^a^	0.001	0.001	0.006
Crude fat	82.48 ± 1.48^b^	88.54 ± 1.77^a^	88.52 ± 1.68^a^	85.18 ± 0.76^ab^	88.42 ± 0.38^a^	0.018	0.061	0.093
Crude fiber	22.08 ± 0.93^b^	33.93 ± 6.58^a^	31.66 ± 2.07^ab^	24.69 ± 1.63^ab^	35.25 ± 1.86^a^	0.048	0.122	0.564
Acid detergent fiber	20.16 ± 7.48	27.12 ± 5.83	39.82 ± 2.39	28.81 ± 3.35	26.01 ± 1.17	0.098	0.377	0.025
Neutral detergent fiber	64.34 ± 1.79	63.22 ± 3.12	64.24 ± 1.47	59.55 ± 1.94	56.32 ± 1.31	0.054	0.008	0.211
Gross energy	75.81 ± 0.56^b^	81.93 ± 1.46^a^	83.77 ± 1.73^a^	81.56 ± 1.10^a^	82.00 ± 0.53^a^	0.003	0.006	0.003

Means (n = 6) within a row with different superscript letters differ significantly (P < 0.05). FRF, fermented ramie feed.

### 3.6 Intestinal morphology

**Figure 1:**
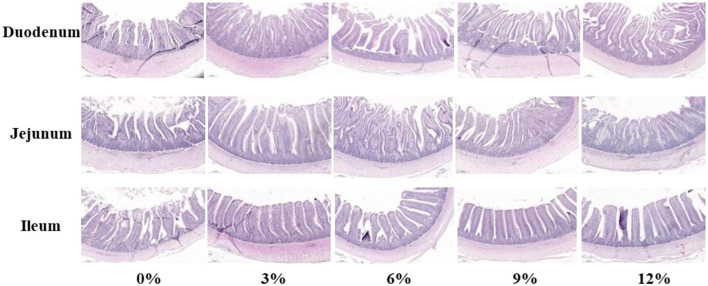
Effect of FRF on intestinal morphology of ducks (40 ×).

As presented in Figure 1 and Table 6, no significant effect in the morphology of jejunum and ileum among groups was found in present study (*P* > 0.05). Compared with the control group, supplemental 3%, 6%, 9%, and 12% FRF significantly improved the VH in the duodenum of ducks (linear and quadratic, *P* < 0.05). Moreover, FRF tended to increase the V/C in the both jejunum (*P* = 0.088) and ileum (*P* = 0.085) of ducks.

**Table 6:** Effect of FRF on intestinal morphology of ducks.

**Items**	**FRF dosage addition**					***P-value***		
	**0**	**3%**	**6%**	**9%**	**12%**	**ANOVA**	**Linear**	**Quadratic**
**Duodenum**								
VH, μm	651.34 ± 14.60^b^	725.69 ± 11.02^a^	730.19 ± 9.04^a^	734.30 ± 13.21^a^	725.01 ± 33.97^a^	0.022	0.014	0.024
CD, μm	232.9135 ± 6.40	242.77 ± 6.92	230.94 ± 6.66	237.56 ± 8.56	239.67 ± 8.65	0.795	0.729	0.917
V/C	2.80 ± 0.08	3.00 ± 0.05	3.17 ± 0.08	3.10 ± 0.08	3.06 ± 0.23	0.305	0.125	0.138
**Jejunum**								
VH, μm	562.44 ± 22.90	659.60 ± 55.42	624.47 ± 52.45	612.46 ± 25.99	571.50 ± 10.56	0.373	0.810	0.085
CD, μm	186.92 ± 8.21	169.52 ± 8.22	171.52 ± 9.61	155.34 ± 12.46	181.84 ± 8.71	0.198	0.429	0.063
V/C	3.04 ± 0.19	3.94 ± 0.36	3.68 ± 0.32	4.07 ± 0.38	3.19 ± 0.15	0.088	0.663	0.016
**Ileum**								
VH, μm	576.00 ± 19.04	627.07± 30.43	592.03 ± 9.29	595.11 ± 18.19	571.48 ± 17.63	0.340	0.525	0.151
CD, μm	162.45 ± 12.76	139.48 ± 5.65	135.19 ± 2.57	161.07 ± 10.79	149.53 ± 10.87	0.173	0.887	0.142
V/C	3.64 ± 0.29	4.52 ± 0.24	4.39 ± 0.09	3.76 ± 0.24	3.94 ± 0.34	0.085	0.839	0.056

Means (n = 6) within a row with different superscript letters differ significantly (P < 0.05). FRF, fermented ramie feed; VH, villus height; CD, crypt depth; V/C, the ratio of villus height to crypt depth.

### 3.7 Gut microbiome

**Figure 2:**
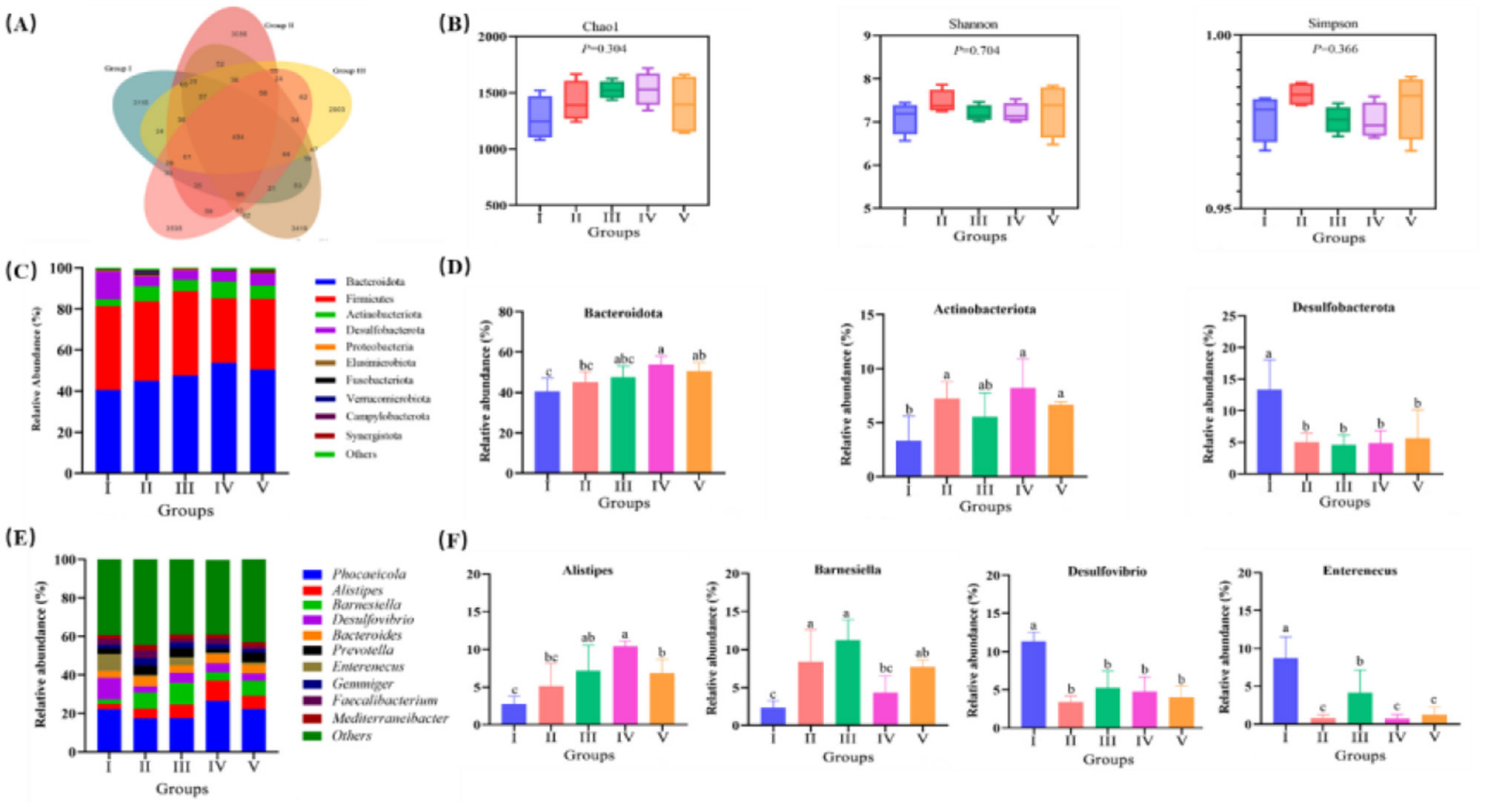
Effect of FRF on intestinal microbiome of ducks. **(A)** Venn diagram. **(B)** Alpha diversity index. **(C)** Relative abundance of cecal microflora at phylum level. **(D)** Significant difference of the abundance of cecal microflora at phylum level. **(E)** Relative abundance of cecal microflora at genus level. **(F)** Significant difference of the abundance of cecal microflora at genus level. Different superscript letters differ significantly (*P* < 0.05).

Across all cecal samples, 21,428 OTUs were detected at a distance level of 0.03 (97% similarity). Specially, 3,115, 3,086, 2,903, 3,419, and 3,535 unique OTUs were identified in the 0, 3%, 6%, 9%, and 12% treatments, respectively (Figure 2A). No significant between-group difference was noted in alpha diversity, as indicated by the Chao1, Shannon, and Simpson (*P* > 0.05; Figure 2B). At the phylum level, the 10 most abundant phyla in cecum were Bacteroidota, Firmicutes, Actinobacteria, Desulfobacterota, Proteobacteria, Fusobacteria, Elusimicrota, Verrucomicrobiota Campylobacter, and Synergistota (Figure 2C). As shown in Figure 2D, compared with the control group, ducks in 9% and 12% groups recorded the higher the relative abundances of Bacteroidota and Actinobacteriota (*P* < 0.05). Moreover, FRF significantly reduced the relative abundance of Desulfobacterota (*P* < 0.05). At the genus level, the 10 most abundant genera were *Phocaeicola, Alistipes, Barnesiella, Desulfovibrio, Bacteroides, Enterenecus, Prevotella, Gemmiger, Faecalibacterium*, and *Mediterraneanbacteria* (Figure 2E). Notably, 6% and 12% FRF supplementation significantly increased the relative abundances of *Alistipes* and *Barnesiella* and reduced the relative abundance of *Desulfovibrio* and *Enterenecus* (*P* < 0.05; Figure 2F).

## 4 Discussion

In animal husbandry, growth metrics serve as key indicators of production efficiency (20). Ramie—a traditional herb—enhances livestock production because of its antibacterial, antioxidant, and anti-inflammatory biological functions (21). A study reported that 12% ramie powder treatment significantly increased the final BW of Yanling white geese, indicating an improvement in growth performance (11). Similarly, a study on *Linwu* ducks revealed improved growth performance, with increased final BW and ADG, after dietary supplementation with ramie powder (10). Li et al. (22) observed improvements in ADG, BW and feed conversion ratio in finishing pigs fed a diet supplemented with ramie. These findings suggest that ramie supplementation enhances growth performance in livestock. On the contrary, no significant effect of ramie was observed in finishing pigs (23) or Boer goats (24). Nevertheless, few studies have investigated the effects of FRF as a partial feed substitute in poultry production. The present study investigated the effects of FRF supplementation on the growth performance of *Linwu* ducks. The results indicated that FRF tended to increase final BW without significantly affecting other growth performance metrics. These variable outcomes may be attributable to differences in poultry breed, ramie processing methods, formulation type, and rearing environments. The immune organ index is a key biomarker of systemic immune function (25). In this study, FRF supplementation increased the thymus index in *Linwu* ducks (i.e., enhanced immune function). This effect may explain the improvement in growth performance.

Serum analytes are vital diagnostic markers of overall physiological status (26). Serum biochemistry serves as an indicator of systemic nutrient metabolism and utilization (27). Serum TG and GLU concentrations are key markers of lipid metabolism and carbohydrate utilization, respectively (28). Other studies were unable to demonstrate that ramie powder supplementation significantly affects serum TG or GLU concentrations (9, 11). Nevertheless, in the present study, FRF supplementation significantly reduced TG and GLU concentrations in ducks. These findings highlight an improvement in energy utilization efficiency. Both antioxidative enzyme activity and peroxide concentrations in serum are reliable biomarkers of *in vivo* antioxidant capacity (29). GSH-Px and CAT, essential components of the antioxidant defense system play vital roles in free radical elimination and oxidative damage prevention (30). A study demonstrated that ramie could improve hepatic SOD activity (9) and T-AOC concentrations in the egg yolk of laying hens (12), thereby enhancing antioxidant capacity. It was also reported by Lin et al. (10) that supplemented ramie powder to the diets of ducks could improve the SOD and GSH activities and upregulate the mRNA expressions of *GSH-Px* in breast and *SOD* in thigh muscle. In addition, ramie supplementation significantly increased T-AOC concentration and reduced the MDA content in the egg yolk (12). The present similarly observed FRF addition significantly improved the CAT and GSH-Px activities in the serum, thereby improving antioxidant capacity. These effects may be attributable to the fact that FRF contains phenolic acids and fermentation-derived prebiotics which can scavenge free radicals and enhance antioxidative enzyme activity.

The morphological integrity of intestinal structures is essential for optimal nutrient digestion and absorption, commonly assessed by measuring VH, CD, and their ratio V/C (31). A higher VH and V/C reflect enhanced epithelial cell turnover and mucosal differentiation, indicating improved digestive and absorptive capacity (32). By contrast, deeper crypts suggest accelerated enterocyte renewal and tissue turnover, which may divert nutrients from growth to intestinal maintenance, potentially impairing growth performance (33). A study reported that ramie powder supplementation significantly increased VH of jejunum and V/C of ileum, thus facilitating intestinal development (9). In the present study, morphological examination revealed that FRF supplementation significantly raised the VH in the duodenum and trended to raise the V/C in the jejunum and ileum of ducks, highlighting improved intestinal morphology. This improvement appears to be closely associated with enhanced antioxidant capacity. The aforementioned benefit is reflected in the digestibility of and metabolic rates for specific nutrients. The apparent metabolic rate of nutrients is a practical indicator for evaluating the efficiency of nutrient utilization in feed (34). In the work, FRF supplementation significantly increased apparent metabolic rate for dry matter, crude protein, crude ash, crude fat, crude fibe, and gross energy, suggesting improved the digestion and metabolism of ducks. These results underscore the benefits of fermentation, which improved feed digestibility. Therefore, FRF improved intestinal morphology to strength the digestion and metabolism of nutrients in ducks; this, in turn, might have a positive influence on growth performance in *Linwu* ducks.

The gut microbiota and host maintain a symbiotic relationship, wherein the host provides a niche for microbial colonization and the microbiota contributes to host nutrition, metabolism, and immune modulation (35). Key microbial metabolites, such as short-chain fatty acids (SCFAs), tryptophan derivatives, and bile acids, play essential roles in regulating host immunity, metabolic homeostasis, and gut function. In poultry production, dietary modifications strongly affect gut microbial composition and influence intestinal health (36). Ashayerizadeh et al. (37) reported that substituting soybean meal with fermented cottonseed meal reduced coliforms and increased lactic acid bacteria in the cecum, improving the productive performance of laying hens. Fermented corn and soybean meal during the peak laying period of laying hens modifies cecal microbiota composition, increasing the abundance of beneficial bacteria such as *Parasutterella, Butyricicoccus*, and *Erysipelotrichaceae* members (38). Besides, a recent study of 16S rRNA sequencing data showed that probiotics-fermented feed substantially improves gut microbial diversity and richness by the boost of key probiotics (e.g., *Ligilactobacillus, Limosilactobacillus*, and *Lentilactobacillus*) while reducing potentially pathogenic bacteria (e.g., *Clostridia-vadinBB60* and *Oscillospira*) (39). Interestingly, Liang et al. (40) found ramie supplementation exerted only minor effects on microbial community composition, even though the abundance of the probiotic bacterium *Roseburia inulinivorans* improved. However, few studies have investigated the effects of fermented ramie on poultry gut microbiota. To the best of our knowledge, the present study is the first to explore the effects of FRF on the gut microbiota of *Linwu* ducks. We demonstrated that FRF supplementation significantly increases the abundance of Bacteroidota (promotes digestion and absorption) and Actinobacteriota (alleviates intestinal inflammation) and reduces the abundance of Desulfobacterota (promotes inflammation). At the genus level, FRF supplementation increases the relative abundances of *Alistipes* and *Barnesiella* and reduces the relative abundances of *Desulfovibrio* and *Enterenecus*. FRF increases beneficial bacterial load and reduces harmful bacterial load. FRF optimizes the gut microbiota structure and supports intestinal health. The effects of FRF may be attributable to the SCFAs and prebiotics produced during fermentation; these metabolites enhance intestinal motility and supply energy for villus development.

## 5 Conclusions

Partial substitution of basal feed with FRF significantly improves serum biochemistry, antioxidant capacity, intestinal morphology, and gut microbiota structure, thereby enhancing nutrient metabolism and improving growth performance to a certain extent. Under the conditions of this study, FRF can be used as a novel feed material with an optimal substitution dose of 3%−6%.

## Data Availability

The data presented in the study are deposited in the National Center for BiotechnologyInformation (NCBI) Sequence Read Archive (SRA) repository, accession number PRJNA1298771.

## References

[1] Du Z Yang F Fang J Yamasaki S Oya T Nguluve D. Silage preparation and sustainable livestock production of natural woody plant. Front Plant Sci. (2023) 14:1253178. 10.3389/fpls.2023.1253178 37746011 PMC10514673

[2] Severo Santos JF Naval LP. Spatial and temporal dynamics of water footprint for soybean production in areas of recent agricultural expansion of the Brazilian savannah (Cerrado). J Clean Prod. (2020) 251:119482. 10.1016/j.jclepro.2019.119482

[3] Cheng Y He J Zheng P Yu J Pu JN Huang ZQ. Effects of replacing soybean meal with enzymolysis-fermentation compound protein feed on growth performance, apparent digestibility of nutrients, carcass traits, and meat quality in growing-finishing pigs. J Anim Sci. (2024) 15:127. 10.1186/s40104-024-01080-x 39261875 PMC11391718

[4] Lv Z Li Z Zhang Z Li G Chen Q Lin Q. Impact of *Lonicera* hypoglauca leaf inclusion on immune and antioxidant responses in geese. Ital J Anim Sci. (2025) 24:521–8. 10.1080/1828051X.2024.2448164

[5] An X Chen J Zhang JY Liao YW Dai LJ Wang B. Transcriptome profiling and identification of transcription factors in ramie (*Boehmeria nivea* L. Gaud) in response to PEG treatment, using illumina paired-end sequencing technology. Int J Mol Sci. (2015) 16:3493–511. 10.3390/ijms16023493 25658800 PMC4346909

[6] Tang S He Y Zhang P Kang J Yan Q Han X. Substitution of ramie (*Boehmeria nivea* I) for alfalfa in improving the carcass and meat quality of Liuyang Black goats. Anim Nutr. (2021) 7:688–94. 10.1016/j.aninu.2020.11.020 34430723 PMC8367831

[7] Wang Q Rehman M Peng DX Liu LJ. Antioxidant capacity and α-glucosidase inhibitory activity of leaf extracts from ten ramie cultivars. Ind Crop Prod. (2018) 122:430–7. 10.1016/j.indcrop.2018.06.020

[8] Sung MJ Davaatseren M Kim SH Kim MJ. Hwang JT. Boehmeria nivea attenuates LPS-induced inflammatory markers by inhibiting p38 and JNK phosphorylations in RAW2647 macrophages. Pharm Biol. (2013) 5:1131–6. 10.3109/13880209.2013.781196 23750815

[9] Wang X Liu Y Zhao HH Wu YM Liu CJ Duan GY. Effects of dietary ramie powder at various levels on the production performance, serum biochemical indices, antioxidative capacity, and intestinal development of laying hens. Front Physiol. (2022) 12:823734. 10.3389/fphys.2021.823734 35242047 PMC8887865

[10] Lin Q Liu Y Wang X Wang YZ Huang P Liu CJ. Effect of dietary ramie powder (*Boehmeria nivea*) at various levels on growth performance, carcass and meat qualities, biochemical indices, and antioxidative capacity of Linwu ducks. Front Physiol. (2022) 13:839217. 10.3389/fphys.2022.839217 35356076 PMC8959830

[11] Chen FM He JY Wang X Lv T Liu CJ Liao LP. Effect of dietary ramie powder at various levels on the growth performance, meat quality, serum biochemical indices and antioxidative capacity of Yanling white geese. Animals. (2022) 12:2045. 10.3390/ani12162045 36009636 PMC9404410

[12] Wang X Peng SM Liu Y Liao S Zhao HH Duan GY. Effect of ramie on the production performance of laying hens, and the quality, nutrient composition, antioxidation of the eggs. Front Physiol. (2022) 13:854760. 10.3389/fphys.2022.854760 35707011 PMC9189287

[13] Hadidi M Aghababaei F Gonzalez-Serrano DJ Goksen G Trif M McClements DJ. Plant-based proteins from agro-industrial waste and by-products: Towards a more circular economy. Int J Biol Macromol. (2024) 261:129576. 10.1016/j.ijbiomac.2024.129576 38253140

[14] Tiwari UP Mandal RK Neupane KR Mishra B Jha R. Starchy and fibrous feedstuffs differ in their in vitro digestibility and fermentation characteristics and differently modulate gut microbiota of swine. J Anim Sci Biotechnol. (2022) 13:53. 10.1186/s40104-022-00699-y 35501888 PMC9063073

[15] Filipe D Vieira L Ferreira M Oliva-Teles A Salgado J Belo I. Enrichment of a plant feedstuff mixture's nutritional value through solid-state fermentation. Animals. (2023) 13:2883. 10.3390/ani13182883 37760283 PMC10525834

[16] Tran TV Kim YS Yun HH Nguyen DH Bui TT Tran PV. A blend of bacillus-fermented soybean meal, functional amino acids, and nucleotides improves nutrient digestibility, bolsters immune response, reduces diarrhea, and enhances growth performance in weaned piglets. J Anim Sci. (2024) 102:skae293. 10.1093/jas/skae293 39320170 PMC11497617

[17] Chi ZH Zhang MQ Fu BT Wang XX Yang H Fang XY. Branched short-chain fatty acid-rich fermented protein food improves the growth and intestinal health by regulating gut microbiota and metabolites in young pigs. J Agric Food Chem. (2024) 72:21594–609. 10.1021/acs.jafc.4c04526 39303156

[18] Reda FM Madkour M Abd El-Azeem N Aboelazab O Ahmed SYA Alagawany M. Tomato pomace as a nontraditional feedstuff: productive and reproductive performance, digestive enzymes, blood metabolites, and the deposition of carotenoids into egg yolk in quail breeders. Poult Sci. (2022) 101:101730. 10.1016/j.psj.2022.101730 35176706 PMC8857486

[19] Zhu X Zhang Y Zhao Y Tao L Liu H Dong W. Effects of dietary supplementation with itaconic acid on the growth performance, nutrient digestibility, slaughter variables, blood biochemical parameters, and intestinal morphology of broiler chickens. Poult Sci. (2022) 101:101732. 10.1016/j.psj.2022.101732 35176702 PMC8851234

[20] Li Z Long L Jin X Li Y Wu Q Chen X. Effects of *Clostridium butyricum* on growth performance, meat quality, and intestinal health of broilers. Front Vet Sci. (2023) 10:1107798. 10.3389/fvets.2023.1107798 36761883 PMC9902377

[21] Rehman M Gang D Liu Q Chen Y Wang B Peng D. Ramie, a multipurpose crop: potential applications, constraints and improvement strategies. Ind Crop Prod. (2019) 137:300–7. 10.1016/j.indcrop.2019.05.029

[22] Li YH Liu YY Li FN Sun A Lin Q Huang XG. Effects of dietary ramie powder at various levels on growth performance, antioxidative capacity and fatty acid profile of finishing pigs. J Anim Physiol Anim Nutr. (2019) 103:564–73. 10.1111/jpn.13031 30549111

[23] Li Y Liu Y Li F Lin Q Dai Q Sun J. Effects of dietary ramie powder at various levels on carcass traits and meat quality in finishing pigs. Meat Sci. (2018) 143:52–9. 10.1016/j.meatsci.2018.04.019 29715660

[24] Wei J Guo W Yang X Chen F Fan Q Wang H. Effects of dietary ramie level on growth performance, serum biochemical indices, and meat quality of Boer goats. Trop Anim Health Prod. (2019) 51:1935–41. 10.1007/s11250-019-01891-5 31134555

[25] Liu YL Yan T Ren ZZ Yang XJ. Age-associated changes in caecal microbiome and their apparent correlations with growth performances of layer pullets. Anim Nutr. (2021) 7:841–8. 10.1016/j.aninu.2020.11.019 34466688 PMC8379648

[26] Spaans OK Kuhn-Sherlock B Hickey A Crookenden MA Heiser A Burke CR. Temporal profiles describing markers of inflammation and metabolism during the transition period of pasture-based, seasonal-calving dairy cows. J Dairy Sci. (2022) 105:2669–98. 10.3168/jds.2021-20883 34998544

[27] Wang J Xiao YX Li JJ Qi M Tan B. Serum biochemical parameters and amino acids metabolism are altered in piglets by early-weaning and proline and putrescine supplementations. Anim Nutr. (2021) 7:334–45. 10.1016/j.aninu.2020.11.007 34258421 PMC8245818

[28] Mitin H Zulkifli I Jamri MHC Zamzuri NA Samian NA Hussein AN. Alleviation of catching and crating stress by dietary supplementation of Bacillus subtilis in Pekin ducks. Animals. (2022) 12:3479. 10.3390/ani12243479 36552400 PMC9774105

[29] Zhao YY Li Z Wang XC Zhao F Wang C Zhang QY. Resveratrol attenuates heat stress-induced impairment of meat quality in broilers by regulating the Nrf2 signaling pathway. Animals. (2022) 12:1889. 10.3390/ani12151889 35892539 PMC9330235

[30] Kakkar R Kalra J Mantha SV Prasad K. Lipid peroxidation and activity of antioxidant enzymes in diabetic rats. Mol Cell Biochem. (1995) 151:113–9. 10.1007/BF01322333 8569756

[31] Suzuki T Aoki K Shimokobe K Omiya S Funayama C Takahashi T. Age-related morphological and functional changes in the small intestine of senescence-accelerated mouse. Exp Gerontol. (2022) 163:111795. 10.1016/j.exger.2022.111795 35378239

[32] Zhang C Wang C Chen K Zhao X Geng Z. Effect of l-theanine on growth performance, intestinal development and health, and peptide and amino acid transporters expression of broilers. J Sci Food Agric. (2020) 100:1718–25. 10.1002/jsfa.10192 31821574

[33] Li Z Jin X Wu Q Long L Li Y Zhang Q. Effects of encapsulated thymol and carvacrol mixture on growth performance, antioxidant capacity, immune function and intestinal health of broilers. Ital J Anim Sci. (2022) 21:1651–9. 10.1080/1828051X.2022.2151944

[34] Noblet J Wu S-B Choct M. Methodologies for energy evaluation of pig and poultry feeds: a review. Anim Nutr. (2022) 8:185–203. 10.1016/j.aninu.2021.06.015 34977388 PMC8685914

[35] Mithieux G. The gut microbiota: stable bioreactor of variable composition. Trends Endocrinol Metab. (2022) 33:443–6. 10.1016/j.tem.2022.04.005 35584972

[36] Rizzetto L Fava F Tuohy KM Selmi C. Connecting the immune system, systemic chronic inflammation and the gut microbiome: The role of sex. J Autoimmun. (2018) 92:12–34. 10.1016/j.jaut.2018.05.008 29861127

[37] Ashayerizadeh A Jazi V Rezvani MR Mohebodini H Soumeh EA Abdollahi MR. An investigation into the influence of fermented cottonseed meal on the productive performance, egg quality, and gut health in laying hens. Poult Sci. (2024) 103:103574. 10.1016/j.psj.2024.103574 38564832 PMC10999706

[38] Liu YL Feng J Wang YM Lv J Li JH Guo LJ. Fermented corn-soybean meal mixed feed modulates intestinal morphology, barrier functions and cecal microbiota in laying hens. Animals. (2021) 11:3059. 10.3390/ani11113059 34827791 PMC8614397

[39] Li Z Li C Lin F Yan L Wu H Zhou H. Duck compound probiotics fermented diet alters the growth performance by shaping the gut morphology, microbiota and metabolism. Poult Sci. (2024) 103:103647. 10.1016/j.psj.2024.103647 38598908 PMC11017063

[40] Liang X Zhai Z Ren F Jie Y Kim S-K Niu K-M. Metagenomic characterization of the cecal microbiota community and functions in finishing pigs fed fermented *Boehmeria nivea*. *Front Vet Sci*. (2023) 10:1253778. 10.3389/fvets.2023.1253778 37841475 PMC10569026

[41] Schloss PD Westcott SL Ryabin T Hall JR Hartmann M Hollister EB. Introducing mothur: open-source, platform-independent, community-supported software for describing and comparing microbial communities. Appl Environ Microbiol. (2009) 75:7537–41. 10.1128/AEM.01541-09 19801464 PMC2786419

[42] Skoien JO Bloeschl G Laaha G Pebesma E Parajka J Viglione A. rtop: An R package for interpolation of data with a variable spatial support, with an example from river networks. Comput Geosci. (2014) 67:180–90. 10.1016/j.cageo.2014.02.009

[43] National Research Council. Nutrient Requirements of Poultry, 9th Edn. National Academy Press (1994).31435969

