# Supplementary material for: Effects of fermented ramie feed on the growth performance, serum biochemistry, metabolic capacity, antioxidant capacity, and intestinal health of Linwu ducks
Source: Front Vet Sci. 2025 Aug 4;12:1646055. doi: 10.3389/fvets.2025.1646055 (PMC12359484; doi:10.3389/fvets.2025.1646055)
Supplement: Supplementary file 1 [file Table_1.docx]

Supplementary Material

## Table S1. Ingredient composition and nutrient content of basal diet.

| **Ingredient, %** |  | **Calculated Nutrient Content** |  |
| --- | --- | --- | --- |
| Corn | 60.00 | Metabolic energy, MJ/kg | 11.50 |
| Soybean meal | 14.49 | Crude protein, % | 16.09 |
| Wheat-middling | 6.81 | Calcium, % | 0.89 |
| Wheat bran | 7.00 | Available phosphorus, % | 0.40 |
| Cottonseed meal | 3.37 | SID^[[1]](#footnote-1)^ lysine, % | 0.90 |
| Rapeseed meal | 4.00 | SID^a^ threonine, % | 0.43 |
| Soybean oil | 0.04 | SID^a^ methionine + cysteine, % | 0.72 |
| Limestone | 1.46 |  |  |
| Dicalcium phosphate | 1.21 |  |  |
| Salt | 0.30 |  |  |
| DL-Methionine | 0.17 |  |  |
| L-Lysine HCl | 0.15 |  |  |
| Non-antibiotic premix^[[2]](#footnote-2)^ | 1.00 |  |  |

**Table S2.** Primers used for Real-Time PCR.

| **Genes** | **GenBank ID** | **Primer sequences（5'—3'）** |
| --- | --- | --- |
| *ZO-1* | XM_015278975.3 | F: TCCCTAAAGGCGAAGAAGTA |
|  |  | R: CAACAATGCGACGATAAACA |
| *occludin* | NM_205128.1 | F: TCCTCATCGTCATCCTGCTCTG |
|  |  | R: CCATCCGCCACGTTCTTCAC |
| *Nrf2* | NM_205117.1 | F: AGTGACCCAGTCTTCATTTC |
|  |  | R: TCTTCCCAAACTTGCTCTAT |
| *GSH-Px* | NM_001277853.3 | F: GGCAAAGTGCTGCTGGTGGTC |
|  |  | R: TCTCCTCGTTGGTGGCGTTCT |
| *SOD1* | NM_205064.2 | F: AAGGGAGGAGTGGCAGAAGT |
|  |  | R: GCTAAACGAGGTCCAGCATT |
| *CAT* | NM_001031215.2 | F: CTTCCTGGTCTTTCTACATTC |
|  |  | R: ATACGCCATCTGTTCTACCT |
| *NF-κB* | NM_001001472.2 | F: ACTTGGCGATCATTCACGAGG |
|  |  | R: AGCGGAGTCTGGCTGAGGTT |
| *IL-6* | NM_204628.1 | F: GAAATCCCTCCTCGCCAATCT |
|  |  | R: CCTCACGGTCTTCTCCATAAACG |
| *IL-10* | NM_001004414.2 | F: GCTGTCACCGCTTCTTCACC |
|  |  | R: TCCCGTTCTCATCCATCTTCTC |
| *IL-1β* | NM_204524.1 | F: GACCAAACTGCTGCGGAGGC |
|  |  | R: CGAAGGACTGTGAGCGGGTGT |
| *TNF-α* | NM_204267.2 | F: TGTTCTATGACCGCCCAGTT |
|  |  | R: TTCAGAGCATCAACGCAAAA |
| *β-actin* | NM_205518.1 | F: TGCGTGACATCAAGGAGAAG |
|  |  | R: GGACTCCATACCCAAGAAAGAT |

*ZO-1*: zonula occludens-1; *Nrf2*: nuclear factor erythroid 2-related factor 2; *GSH-Px*: glutathione peroxidase; *SOD1*: superoxide dismutase 1; *CAT*: catalase; *NF-κB*: nuclear factor kappa B; IL: interleukin; *TNF-α*: tumor necrosis factor-α.

1. Standardized ileal digestible. [↑](#footnote-ref-1)
2. Non-antibiotic premix included per kilogram of diet: 12000 IU vitamin A, 2500 IU vitamin D_3_, 20 mg vitamin E, 3 mg vitamin K, 3 mg vitamin B_1_, 8 mg vitamin B_2_, 7 mg vitamin B_6_, 0.03 mg vitamin B_12_, 1.5 mg folic acid, 50 mg nicotinamide, 0.1 mg biotin, 20 mg pantothenic acid, 100 mg Fe (FeSO_4_·H_2_O), 9 mg Cu (CuSO_4_·5H_2_O), 100 mg Mn (MnSO_4_·H_2_O), 110 mg Zn (ZnSO_4_·H_2_O), 0.6 mg I (KI), 0.16 mg Se (Na_2_SeO_3_). [↑](#footnote-ref-2)
